# Efficient Mutagenesis of Marek’s Disease Virus-Encoded microRNAs Using a CRISPR/Cas9-Based Gene Editing System

**DOI:** 10.3390/v12040466

**Published:** 2020-04-20

**Authors:** Jun Luo, Man Teng, Xusheng Zai, Na Tang, Yaoyao Zhang, Ahmedali Mandviwala, Vishwanatha R. A. P. Reddy, Susan Baigent, Yongxiu Yao, Venugopal Nair

**Affiliations:** 1 The Pirbright Institute & UK-China Centre of Excellence for Research on Avian Diseases, Pirbright, Ash Road, Guildford, Surrey GU24 0NF, UK; tm135@aliyun.com (M.T.); monkey2006zxs@163.com (X.Z.); tangna0543@163.com (N.T.); zhangyaoyao3848@126.com (Y.Z.); ahmedali.mandviwala@gmail.com (A.M.); vishi.reddy@pirbright.ac.uk (V.R.A.P.R.); sue.baigent@pirbright.ac.uk (S.B.); yongxiu.yao@pirbright.ac.uk (Y.Y.); 2 Key Laboratory of Animal Immunology, Ministry of Agriculture & Henan Provincial Key Laboratory of Animal Immunology, Henan Academy of Agricultural Sciences, Zhengzhou 450002, China; 3 UK-China Centre of Excellence for Research on Avian Diseases, Henan Academy of Agricultural Sciences, Zhengzhou 450002, China; 4 Ministry of Education Key Lab for Avian Preventive Medicine, Yangzhou University, Yangzhou 225009, China; 5 Binzhou Animal Science and Veterinary Medicine Academy & UK-China Centre of Excellence for Research on Avian Diseases, Binzhou 256600, China; 6 College of Animal Science and Technology, Guangxi University, Nanning 530004, China

**Keywords:** CRISPR, herpesvirus, Marek’s disease virus, miRNA, gene editing

## Abstract

The virus-encoded microRNAs (miRNAs) have been demonstrated to have important regulatory roles in herpesvirus biology, including virus replication, latency, pathogenesis and/or tumorigenesis. As an emerging efficient tool for gene editing, the clustered regularly interspaced short palindromic repeat (CRISPR)/Cas9 system has been successfully applied in manipulating the genomes of large DNA viruses. Herein, utilizing the CRISPR/Cas9 system with a double-guide RNAs transfection/virus infection strategy, we have established a new platform for mutagenesis of viral miRNAs encoded by the Marek’s disease virus serotype 1 (MDV-1), an oncogenic alphaherpesvirus that can induce rapid-onset T-cell lymphomas in chickens. A series of miRNA-knocked out (miR-KO) mutants with deletions of the Meq- or the mid-clustered miRNAs, namely RB-1B∆Meq-miRs, RB-1B∆M9-M2, RB-1B∆M4, RB-1B∆M9 and RB-1B∆M11, were generated from vvMDV strain RB-1B virus. Interestingly, mutagenesis of the targeted miRNAs showed changes in the in vitro virus growth kinetics, which is consistent with that of the in vivo proliferation curves of our previously reported GX0101 mutants produced by the bacterial artificial chromosome (BAC) clone and Rec E/T homologous recombination techniques. Our data demonstrate that the CRISPR/Cas9-based gene editing is a simple, efficient and relatively nondisruptive approach for manipulating the small non-coding genes from the genome of herpesvirus and will undoubtedly contribute significantly to the future progress in herpesvirus biology.

## 1. Introduction

MicroRNAs (miRNAs), one of the most important types of RNA, are endogenous small (approximately 22–24 nt) non-coding RNAs. In the past two decades, thousands of miRNAs have been characterized, identified and/or annotated in the genomes of 271 organisms [1], including humans, metazoans, plants and some lower eukaryotes. These small molecules have been demonstrated to play critical post-transcriptional regulatory roles through the RNA-induced gene silencing in various biological processes, such as cellular development, differentiation, disease progress and all aspects of cancer biology [2,3]. Interestingly, hundreds of viral miRNAs have also been discovered in a variety of viral genomes [1], including oncogenic herpesviruses such as Epstein–Barr virus (EBV) and Kaposi’s sarcoma-associated herpesvirus (KSHV). The latest advances in viral miRNA biology have displayed their important functions involved in virus replication, latent infection, pathogenesis and/or tumorigenesis [4,5,6,7].

**Figure 1:**
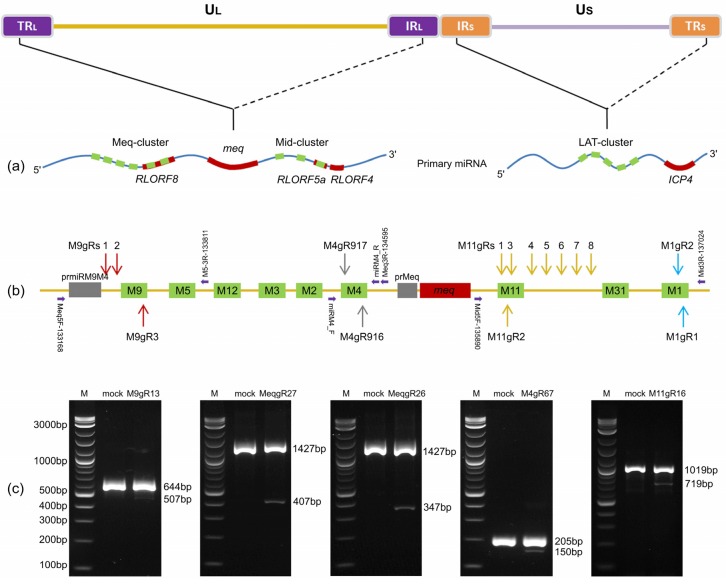
Schematics demonstrating the single guide RNA (sgRNA) targeting sites and PCR analysis of corresponding gene editing of Marek’s disease virus serotype 1 (MDV-1) microRNAs (miRNAs). (**a**) The viral genome and primary transcripts of miRNA clusters. The relative genomic locations of miRNA clusters are shown by solid and dashed lines [17]. UL and US, unique long or unique short regions; TRL and IRL, terminal or internal repeat long regions; IRS and TRS, internal or terminal repeat short regions. Precursor miRNAs (e.g., miR-M4) are abbreviated (M4) and are shown by green blocks while the open reading frames of protein-coding genes are shown by red blocks. (**b**) Relative genomic location of sgRNAs (gRs). The gRNAs targeting the same miRNA are shown by arrows in same colors. Horizontal arrows in violet indicate the primer locations and directions used for detection PCR. (**c**) PCR analysis of the mutated miRNA genomic regions mediated by different gRNA combinations.

A large number of viral miRNAs have been recently reported in the genomes of avian herpesvirus species [8,9,10,11,12], including all the three serotypes of Marek’s disease virus, MDV-1, MDV-2, and MDV-3 (herpesvirus of turkeys, HVT), which have been reclassified as Gallid herpesvirus 2 (GaHV2), Gallid herpesvirus 3 (GaHV3), and Meleagrid herpesvirus 1 (MeHV1), respectively [13]. As an aggressively oncogenic herpesvirus, MDV-1 induces rapid-onset T lymphomas in its natural host chickens, along with paralysis and immunosuppression, conditions collectively named as Marek’s disease (MD) [14]. Historically, MD is regarded as an excellent model for the study of cancer biology [15], and the first successful example of the use of vaccinations against a cancer [16]. MDV-1 genome expresses a total of 26 mature miRNAs, encoded in 14 precursors as illustrated in Figure 1a, in three gene clusters named as the Meq-, mid- and LAT-clusters within the inverted repeat regions of the genome [8,11,17]. Several MDV-1 miRNAs, such as the viral miR-155 ortholog miR-M4-5p [18,19,20,21,22,23,24], miR-M3-5p [25], and miR-M7-5p [26], have been demonstrated to play key roles in MDV life cycle to regulate herpesvirus DNA cleavage/packaging, latency or especially virally-induced tumorigenesis. While it is known that most of the viral miRNA functions are related to the pathogenicity and/or oncogenicity [27,28], underlying molecular regulatory mechanisms mediated by these tiny RNAs in MDV biology still remain unclear.

The clustered regularly interspaced short palindromic repeat (CRISPR)-Cas system is an adaptive immune mechanism for bacteria and archaea to protect against the attacks of viruses or other invading genetic elements [29,30,31,32]. Based on the type II CRISPR-Cas system consisting of a single guide RNA (sgRNA), a trans-activating crRNA (tracrRNA) and a RNA-guided endonuclease Cas9 derived from Streptococcus pyogenes, the latest developed CRISPR/Cas9 gene editing technique has been displayed as a simple, efficient and powerful tool for gene editing in biological research [33,34,35]. It has not only been widely applied for generating gene knockout (KO) cell lines and animal models, but has also been used for manipulating large viral DNA genomes, such as Epstein–Barr virus (EBV) [36], herpes simplex virus type I (HSV-1) [32,37,38], guinea pig cytomegalovirus (GPCMV) [39], pseudorabies virus (PrV) [40,41,42], vaccinia virus (VACV) [43,44], and duck enteritis virus (DEV) [45,46]. We have successfully demonstrated the manipulation of the HVT and MDV-1 genomes using the CRISPR/Cas9 system both for generating recombinant vaccines and for studying the viral protein-coding gene functions respectively [47,48,49,50,51].

In the present work, we have applied the double-sgRNA mediated gene editing strategy to investigate whether the viral miRNAs encoded in the MDV-1 genomes can be efficiently manipulated using the same system. We found that both of the entire viral miRNA gene clusters and the single miRNA genes encoded in the Meq- or mid-clusters can be deleted from the targeted genomic regions to generate miRNA-knocked out (miR-KO) mutant viruses, which can be further confirmed by PCR analysis and DNA sequencing. The mutants were further analyzed by immunofluorescent s assay (IFA) and determination of the in vitro virus growth kinetics. Our results show that the CRISPR/Cas9-based gene editing is a simple, rapid, and efficient approach to manipulate the MDV genome for a better understanding of the regulatory roles of small non-coding RNAs in MDV biology.

## 2. Materials and Methods

### 2.1. Cells and Virus

Primary chicken embryo fibroblast (CEF) monolayers prepared from 10-day-old specific pathogen free (SPF) embryos were maintained in M199 medium (Thermo Fisher Scientific, Basingstoke, United Kingdom) supplemented with 5% fetal calf serum (FCS, Sigma, Gillingham, United Kingdom), 10% tryptose phosphate broth (TPB, Sigma, St. Louis, MO, USA), 100 units/mL of penicillin and streptomycin (Thermo Fisher Scientific, Paisley, Scotland, UK) and 0.25 mg/mL Fungizone (Sigma, St. Louis, MO, USA). The cells were kept at 38.5 °C in 5% CO_2_ incubators. MDV-1 strain RB-1B was used as a parental virus for constructing miRNA-KO mutants.

### 2.2. Construction of sgRNAs Plasmids

The gRNAs targeting the viral miRNAs in RB-1B were designed using the online CRISPR sgRNA Design Tool and CHOPCHOP [52,53]. The oligonucleotides corresponding to the gRNAs upstream or downstream to target miRNA genes were synthesized (Integrated DNA Technologies, Leuven, Belgium) and cloned into the Bbs I restriction sites of CRISPR/Cas9 vectors pX330A-1×2 (Addgene plasmid ID: 58766) and pX330S-2 (Addgene plasmid ID: 58778), namely as pX330A-gR or pX330S2-gR, respectively. For constructing the double-gRNA plasmids, the downstream gRNAs were digested from pX330S-2-gRNA plasmids and cloned into the Bsa I restriction sites of pX330A-1×2-gRNA plasmids, namely as pX330A-dgR. The gRNA targets and oligo nucleotides used for making guide RNA plasmids are listed in Tables S1 and S2, respectively.

### 2.3. Generation of Viral miRNA Mutated RB-1B Viruses

The confluent primary CEF monolayers in 24-well plates were co-transfected with different combinations of pX330A-gR and pX330S-2-gR plasmids targeting to distinct miRNA regions listed in Table S1, using Lipofectamine^®^ Reagent (Thermo Fisher Scientific, Basingstoke, United Kingdom) according to the manufacturer’s instruction. At 24 h post-transfection (hpt), the CEF cells were infected with RB-1B at 0.01 pfu/cell. The infected CEFs were trypsinized two days later, half for PCR analysis using the primers listed in Table S3 (#1-4) and the other half were partially passed into CEF monolayers in 6-well plates to produce individual plaques. At 2–3 days post passage, the single virus plaques were picked into 24-well plates with fresh CEF monolayers, followed by PCR analysis, virus purification and further passages.

### 2.4. Characterization of miR-KO RB-1B Viruses

The CEF monolayers in 24-well plates were infected with parental RB-1B or the purified single plaques of RB-1B∆miR mutants. The infected cells were collected at 48 h post-infection (hpi) and lysed in 1 × squishing buffer (10 mM Tris-HCl, pH 8, 1 mM EDTA, 25 mM NaCl, and 200 μg/mL Proteinase K) at 65 °C for 30 min. PCR was performed to identify the expected miR-KO viruses using the corresponding primers outside the targeted sites (Table S3, #1-4), and then the mutated PCR products were purified for further DNA sequencing.

### 2.5. Immunofluorescence Assay (IFA)

The expressions of viral proteins Meq, gB, and pp38 in CEF cells infected with the RB-1B miR-KO mutants were determined by IFA using inverted immunofluorescence microscopy as described previously [48,49]. For IFA analysis, the rabbit anti-Meq polyclonal antibody and MDV-1 specific mAbs HB3 and BD1 were used as primary antibodies, and the Alexa Fluor^TM^ 568 Goat anti-Rabbit IgG (H+L) and 488 Rabbit anti-Mouse IgG (H+L) (Invitrogen) were used as secondary antibodies for staining the expression of Meq, gB, and pp38 proteins, respectively. Images were taken on DM IRB Inverted Microscope (Leica Microsystems, Wetzlar, Germany).

### 2.6. qRT-PCR Analysis for MDV-1 miRNA Expressions

The relative expression levels of miR-M4-5p, miR-M9-5p, miR-M11-5p, miR-M12-3p, and miR-M31-3p in RB-1B or miR-KO virus infected CEFs were determined by qRT-PCR as previously described [49]. The TaqMan MicroRNA Assay System (Thermo Fisher Scientific, Basingstoke, United Kingdom) was used for reverse transcription reaction, each of which was performed twice independently using 10 ng total RNA as a template. PCR was run in triplicate on the ABI Prism 7500 Sequence Detection System. The detailed oligonucleotide primers and labeled TaqMan probes are listed in Table S4. All values were normalized to miR-M31-3p, a viral miRNA in the mid-cluster with moderate expression level, and the relative expression levels were calculated as fold changes compared to those from parental RB-1B virus-infected cells.

### 2.7. The Growth Kinetics of the miR-KO RB-1B Viruses

The in vitro growth kinetics of the parental and miR-KO RB-1B viruses on CEF cells were determined by qPCR at different time points post virus infection. For each MDV strain, 100 PFU viruses were inoculated into one well of CEF monolayers in 6-well plates. At 24, 48, 72, 96, and 120 hpi, infected cells were collected for DNA extraction using the DNeasy 96 Blood and Tissue Kit (Qiagen, Manchester, United Kingdom). The viral copies were determined by a real-time qPCR analysis to produce the growth curves of the viruses as described previously [54,55]. Briefly, a duplex real-time qPCR was carried out to detect the MDV-1 US2 gene and the chicken ovotransferrin (OVO) gene to enable calculation of MDV genome copies per 10,000 cells. Ten-fold dilution series of DNA extracted from either CVI988-infected CEF cells or non-infected CEF cells were used to produce standard curves for the US2 reaction and the OVO reaction, respectively. MDV genome copies per 10,000 cells were plotted against time points post-infection for each miR-KO virus. Statistically, the differences of viral copies between the parental RB-1B virus and its mutants were compared and analyzed by one-way analysis of variance (one-way ANOVA, LSD) and were considered significant at a probability level of *p* < 0.05.

### 2.8. Growth Kinetics of RB-1B∆M11 Virus in CEFs Overexpressing miR-M11

The RCAS system [56] was chosen for a better overexpression of miR-M11 in CEF cells. The miR-M11 precursor sequence was cloned into the Not I restriction sites of the vector RCAS-B-GFP-CMV to make the miRNA-expressing plasmid, namely RCAS-B-GFP-CMV-M11. In 6-well plates, the confluent CEF cells were transfected with plasmids RCAS-B-GFP-CMV or RCAS-B-GFP-CMV-M11 (200 ng each) 3 days before virus infection and then the miR-M11 overexpressed CEFs were infected with RB-1BΔmiR-M11 while the mock transfected CEFs were separately infected with RB-1B or RB-1BΔmiR-M11 viruses (1000 PFU each per well). At 24, 48, 72, 96, and 120 hpi, infected cells were collected for DNA and miRNA extraction using the DNeasy 96 Blood and Tissue Kit or the miRNeasy Mini Kit (Qiagen, Manchester, United Kingdom), respectively. The viral copies for producing the growth curves of the viruses and the relative expression levels of miR-M11-5p and miR-M12-3p were separately determined by the real-time qPCR or qRT-PCR analysis as described above. For each virus, the experiments were repeated independently in triplicate and statistical analysis was performed as described above.

## 3. Results

### 3.1. The Efficacy of sgRNAs for Editing the Viral miRNA Using CRISPR/Cas9 System

To establish a platform for mutating the viral miRNAs encoded in MDV-1 genomes using CRISPR/Cas9 system, we first designed a series of sgRNAs targeting the corresponding genomic loci of the Meq-clustered miRNAs. As illustrated in Figure 1a,b, three sgRNAs for miR-M9 (M9gRs 1, 2 and 3) and two sgRNAs for miR-M4 (M4gRs 916 and 917) were designed using the online gRNA design tools according to the protocols and rules suggested by Zhang’s lab [56]. Using these sgRNAs, we produced four distinct CRISPR/gRNA plasmids with paired gRNAs, such as pX330-M9gR13 (M9gR1 plus M9gR3), pX330-M4gR67 (M4gR916 plus M4gR917), pX330-MeqgR26 (M9gR2 plus M4gR916), and pX330-MeqgR27 (M9gR2 plus M4gR917), for knocking out the single miRNA genes (miRM9 and miRM4), the entire or the truncated Meq-clusters (Meq-miRs and miRM9-M2), respectively. Based on a co-transfection and virus infection strategy, the gene editing efficacies of these gRNA combinations targeting to RB-1B miRNA genes were tested and the result showed that all the four CRISPR/gRNA plasmids worked efficiently. Compared to the parental RB-1B virus, as demonstrated in Figure 1c, additional smaller bands of PCR products were observed as expected in length for each targeted site.

For the viral miRNAs in the mid-cluster, as illustrated in Figure 1b, a series of sgRNAs targeting miR-M11 (M11gRs #1-8) and miR-M1 (M1gRs 1 and 2) were also designed. For the miR-M11, the CRISPR/gRNA plasmid pX330-M11gR16 (M11gR1 plus M11gR6) demonstrated an efficient mutagenesis of RB-1B virus (Figure 1c), similar to the other sgRNA combinations of pX330-M11gR35 (M11gR3 plus M11gR5) and pX330-M11gR36 (M11gR3 plus M11gR6).

### 3.2. Cloning and Purification of the Viral miRNA-Deletion Mutant RB-1B Viruses

Based on confirmation of the mutagenesis of viral miRNAs mediated by CRISPR/gRNAs, the CEF cells containing mutated RB-1B viruses were trypsinized and transferred to fresh CEF monolayers to produce single viral plaques. For the five targeted mutations to delete the entire Meq-cluster (Meq-miRs), the truncated Meq-cluster (miR-M9-M2), miR-M4, miR-M9, and miR-M11, totals of 72, 60, 72, 96 or 96 single viral plaques were picked, passaged and further identified by PCR analysis with positive mutation rates of 2.8% (2/96), 1.7% (1/60), 6.9% (5/72), 2.1% (2/96), and 2.1% (2/96), respectively. For the first-round detection of RB-1B mutants, PCR products amplified from all the viral DNA samples contained only the smaller bands.

**Figure 2:**
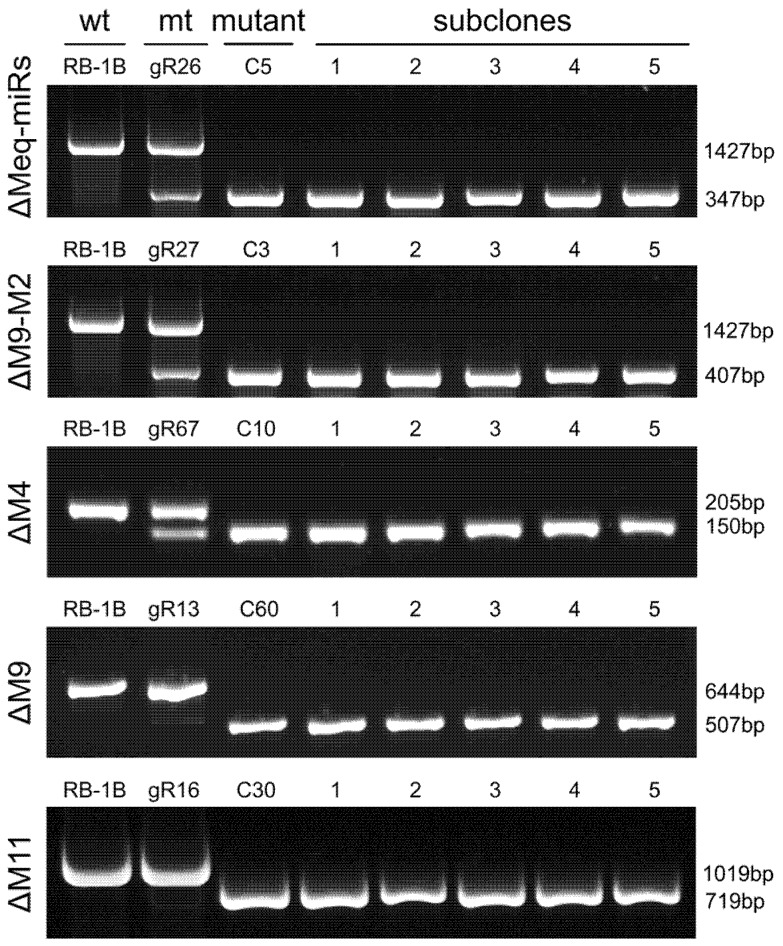
PCR analysis of the mutagenesis in RB-1B viral genome with miRNA-deletions. Wt, wild type; mt, mutated type. The subclones are second round-purified single virus plaques of corresponding RB-1B mutants.

**Figure 3:**
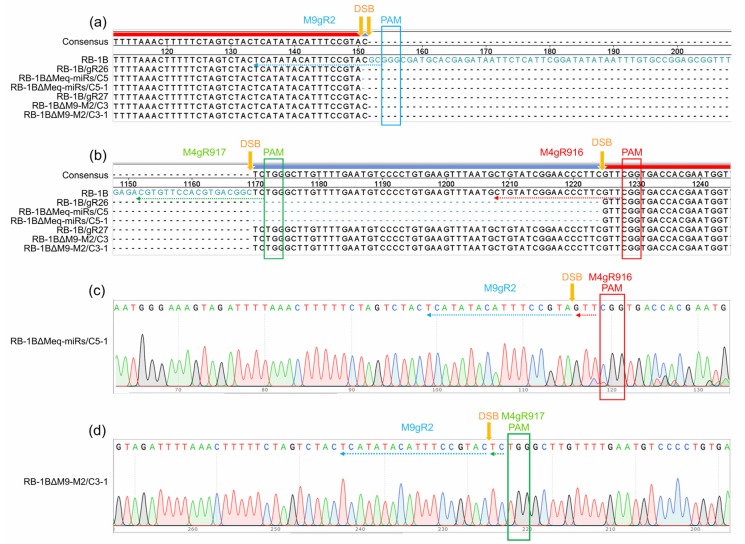
Sequence alignment and identification of the gRNA-mediated Meq-clustered miRNA mutagenesis in RB-1B viral genome. (**a**,**b**) Alignment of the miRNA genes in primarily mutated RB-1B and purified progeny mutants. (**c**,**d**) Demonstration of the double strand breaks (DSBs) in the targeted miRNA genes. The entire or broken gRNA sequences and protospacer adjacent motifs (PAMs) are shown by same colored arrows or square frames, respectively.

Five RB-1B mutants including RB-1B∆Meq-miRs (clone C5), RB-1B∆M9-M2 (clone C3), RB-1B∆M4 (clone C10), RB-1B∆M9 (clone C60), and RB-1B∆M11 (clone C30), were subcloned again to produce single virus plaques for further purification of miR-KO mutants. As expected, all the five randomly picked subclones for each miR-KO mutant contained only the smaller PCR bands (Figure 2). The PCR products amplified from the miR-KO RB-1B subclones C5-1, C3-1, C10-1, C60-1, and C30-1 were gel-extracted for DNA sequencing. Sequence alignment analysis showed that all targeted miRNAs were precisely cut by the nuclease Cas9 at the predicted sites, 2-3 nucleotides upstream of the protospacer adjacent motifs (PAMs) and were omitted from the RB-1B viral genomes (Figure 3 and Figure S1). Thus, all of the miR-KO mutants, including RB-1B∆Meq-miRs (C5-1), RB-1B∆M9-M2 (C3-1), RB-1B∆M4 (C10-1), RB-1B∆M9 (C60-1), and RB-1B∆M11 (C30-1), were passaged and expanded for making the virus stocks for further analysis.

### 3.3. Effects of miR-KO on the Expression of MDV Protein-Coding and Non-Coding Genes

**Figure 4:**
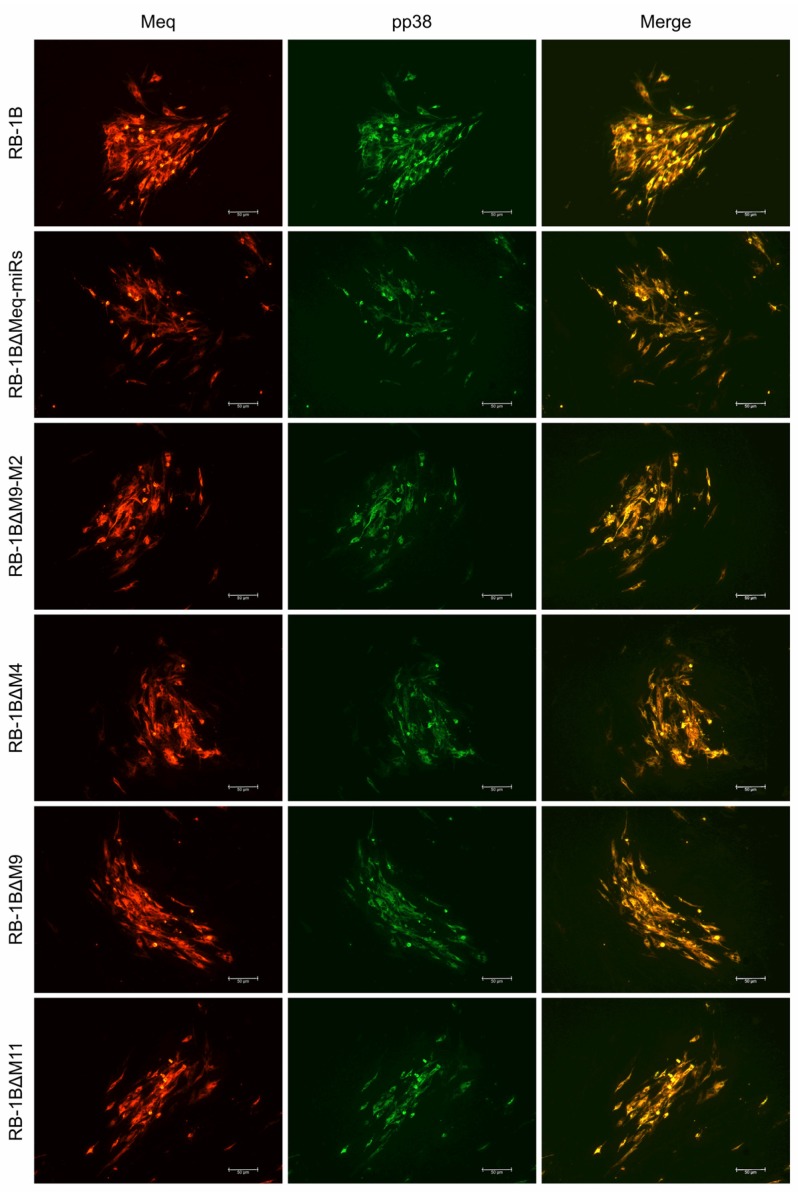
Immunofluorescence assays for detection of viral protein expressions in chicken embryo fibroblast (CEF) infected with RB-1B mutant viruses. Meq, Marek’s EcoQ-encoded protein; pp38, phosphoprotein 38. Scale bar = 50 μm.

**Figure 5:**
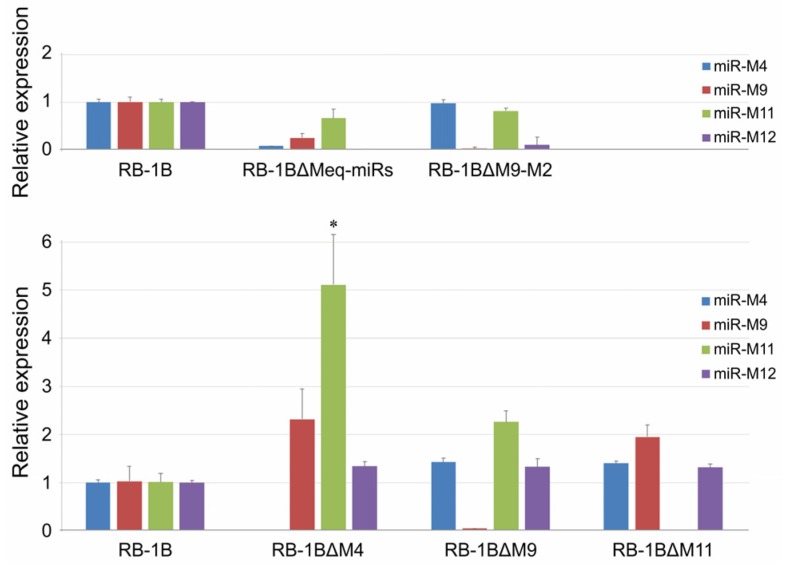
Relative expression levels of miRNAs in parental or mutated RB-1B-infected CEF cells. Asterisk (*) indicates statistically significant difference between miRNA-knocked out (miR-KO) mutant virus and parental RB-1B. *, *p* < 0.05.

The CEF monolayers were infected with the miR-KO viruses and the virus plaques on the infected CEFs were examined by IFA with the polyclonal antibody against MDV-specific Meq protein and the monoclonal antibodies BD1 (MDV-pp38-specific) or HB3 (MDV-gB-specific) sequentially. As expected (Figure 4 and Figure S2), all the three viral proteins Meq, pp38, and gB were expressed as demonstrated by the staining pattern with the specific antibodies in the plaques produced by either the parental RB-1B or miR-KO mutant viruses. For further confirmation of the deletion of viral miRNAs and evaluation of the expression of adjacent genes in virus-infected CEFs, quantitative RT-PCR was performed to analyze the relative expression levels of the randomly selected viral miRNAs from both of the Meq-cluster (miR-M4, miR-M9, and miR-M12) and the mid-cluster (miR-M11 and miR-M31). Compared to the parental RB-1B virus, as shown in Figure 5, all of the selected miRNAs were expressed at normal levels in the miR-KO virus-infected CEFs except for that of the deleted single or clustered miRNAs and the significantly increased miR-M11 expression in miR-M4-KO mutant group (*p* < 0.05). These data not only further confirmed the successful deletion of the specific miRNAs from RB-1B viral genome, but also demonstrated that this targeted deletion did not affect the expression of adjacent genes.

### 3.4. Growth Kinetics of the miR-KO Mutant Viruses

**Figure 6:**
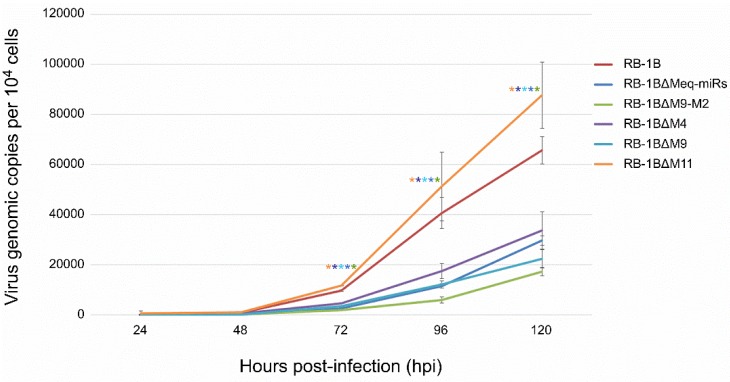
In vitro proliferation of the RB-1B mutant viruses with miRNA-deletions. Viral genomic copies of the parental or mutant viruses per 10^4^ cells, estimated based on MDV-1 US2 gene, were determined by a real-time qPCR on DNA from virus-infected CEFs sampled at different time points post-infection. All the experiments were repeated in triplicate. Asterisk (*) indicates statistically significant differences between miR-KO mutant virus and parental RB-1B at different time points. *, *p* < 0.05.

The potential biological effects of the miRNA deletions on the viruses were also evaluated by comparing the growth kinetics of the miR-KO mutants and the parental RB-1B virus. For this purpose, the CEF monolayers were infected with identical doses of either the parental or miR-KO RB1B viruses. At 24 h intervals for five consecutive days, DNA extracted from the virus-infected cells was used for a quantitative PCR analysis to determine the virus growth curves. Surprisingly, the qPCR data on samples from various time points post-infection showed that the deletions of viral miRNAs from the Meq-cluster and the mid-cluster had inverse effects on virus replication in CEF cells. As demonstrated in Figure 6, the viral copies of RB-1B∆Meq-miRs, RB-1B∆M9-M2, RB-1B∆M4, and RB-1B∆M9 in CEFs at time points of 72–120 hpi were all significantly lower than that of the wild type RB-1B virus, while the copy number of the RB-1B∆M11 virus was significantly higher than the parental RB-1B virus. This data suggested that the deletion of the Meq-clustered miRNAs reduced the in vitro RB-1B replication, while the deletion of miR-M11 in the mid-clustered miRNAs improved virus replication.

### 3.5. Compensatory miR-M11 Overexpression Suppresses the Improved Growth of RB-1B∆M11 Virus

**Figure 7:**
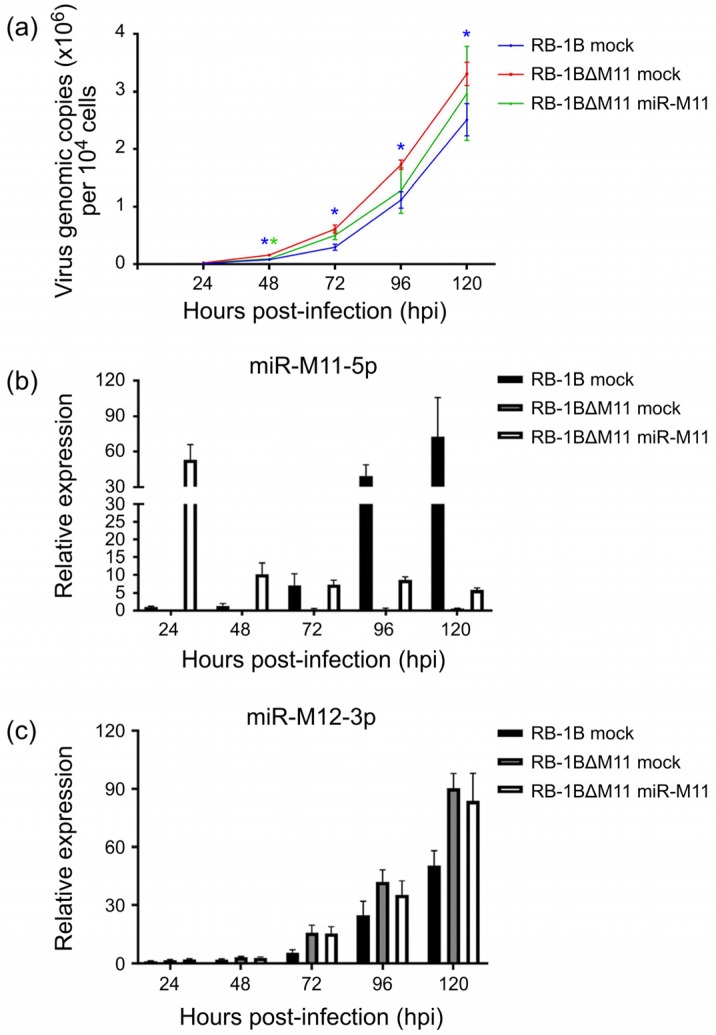
Growth curves of the RB-1BΔM11 virus and miRNA expression levels in miR-M11 overexpressed CEF cells. (**a**) Growth curves of the RB-1B and RB-1BΔM11 viruses in mock or miR-M11 overexpressed CEF cells. The viral copy numbers per 10^4^ cells, based on MDV-1 US2 gene, were determined by a real-time qPCR analysis. Asterisk (*) indicates statistically significant differences of the parental RB-1B or RB-1BΔM11 mutant viruses in miR-M11-overexpressing CEFs compared to that of RB-1BΔM11 in normal CEFs. *, *p* < 0.05. (**b**,**c**) Relative expression levels of miR-M11-5p and miR-M12-3p in the virus-infected normal or miR-M11-overexpressing CEF cells. All the experiments were repeated independently in triplicate.

In order to confirm the direct involvement of miRNAs on the observed changes of virus growth kinetics, we chose the RB-1B∆M11 virus as an example to perform a compensatory overexpression of miR-M11 in CEFs to evaluate the growth of the RB-1B∆M11. As demonstrated in Figure 7a, the viral copy numbers of RB-1BΔM11 virus in mock CEFs from 48 to 120 hpi were significantly higher (*p* < 0.01) than that of the parental RB-1B virus. However, the growth kinetics of RB-1BΔM11 virus in CEFs overexpressing miR-M11 at 48 hpi was significantly slower (*p* < 0.01) than that in the normal CEFs. During the time period from 72 to 120 hpi, RB-1BΔM11 virus was still kept at a lower level although this was not statistically significant. In accordance with the virus replication curves, we also found that the expression levels of miR-M11-5p, a mature miRNA processed from miR-M11, increased significantly from 24 to 120 hpi in the parental RB-1B virus-infected cells. However, in the CEFs overexpressing miR-M11, miR-M11-5p decreased during the same time period (Figure 7b). We also demonstrated that miR-M11-5p continued to be expressed in the miR-M11-overexpressing CEFs although its expression levels from 48 to 120 hpi were much lower than that at 24 hpi. In RB-1BΔM11 infected mock CEFs, no expression of miR-M11-5p was detected during the whole period of the experiment. As for the other reference viral miRNA, in Figure 7c, the expression levels of miR-M12-5p in CEFs had the same tendency as that of the virus growth curves. All the present data, from both the virus growth kinetics and the viral miRNA expressions, confirmed that the deletion of miR-M11 improves virus replication but it could be reversely suppressed by the overexpression of the corresponding miRNAs in virus-infected cells.

## 4. Discussion

In the last two decades, the bacterial artificial chromosome (BAC) clone and Rec E/T homologous recombination techniques [57,58] have been successfully and widely used for manipulating the large DNA viral genome for viral gene function study and for the production of recombinant vaccine viruses. As for the avian herpesviruses, such as MDV-1, MDV-2, and HVT, the BAC mutagenesis has also contributed a lot to editing the viral genomes [59,60,61,62]. In previous studies, we have also produced a series of miRNA-deleted MDV-1 mutants using a GX0101 BAC clone for studying the virus–host interactions mediated by viral miRNAs [21,22,27,28]. However, some of the shortcomings of the BAC engineering approach such as the time-consuming step of cloning of large viral genomes into BAC plasmids, the low frequency of expected homologous recombination, and the insertion of BAC plasmid elements or drug selection markers into the viral genome that sometimes interferes with viral functions, have limited its use. For the recombinant vaccine strains for field use, artificially-inserted marker genes also need to be deleted for regulatory approvals. Moreover, we also found that the residual sequences such as the flipase recombination target (FRT) site left over in viral genomes during the recombination step make the screening and identification of shorter deletions such as those in miR-KO mutants difficult, because of the similar sizes between the FRT sites and targeted miRNA regions.

In order to overcome some of these drawbacks and to establish a simpler and efficient platform for the mutagenesis of tiny genes such as miRNAs encoded by herpesviruses, we have presently applied the CRISPR/Cas9 system to edit a series of viral miRNAs in the oncogenic MDV-1 genome. Based on a transfection/infection strategy with double-guide RNAs, we successfully generated five independent miR-KO mutant viruses, RB-1B∆Meq-miRs, RB-1B∆M9-M2, RB-1B∆M4, RB-1B∆M9, and RB-1B∆M11. We have found that except for the miR-M4-KO mutant, either deletion of the entire miRNA cluster or the other single miRNA genes does not affect the expression of adjacent non-coding genes or representative protein-coding genes, as confirmed by the immunofluorescent staining and qRT-PCR analysis. A previous study [63] has shown that the transcription of the Meq- and the mid-cluster miRNAs gives two distinct transcriptional patterns: during the latent phase of infection the two clusters are driven by the single prmiRM9M4 promoter while during the lytic phase they are transcribed separately using independent promoters, and most of the miRNAs represent two different expression profiles in the different stages of disease progression [64,65]. For the miR-M4-KO mutant, we have observed that once the miR-M4 is omitted from the viral genome, the expression levels of miR-M11 in virus-infected CEFs showed an unusual increase, which greatly strengthened our previous suggestions that the oncogenic or tumor suppressive roles for miR-M4 and miR-M11, respectively, are important to keep a balance during the latency infection and/or MD tumorigenesis [27,28]. Whether miR-M4 participates directly in the regulation of miR-M11 expression in MDV infection deserves to be studied further.

Interestingly, the in vitro viral growth kinetics of CRISPR/Cas9-edited miR-KO viruses in CEF cells demonstrated that, compared to the parental RB-1B virus, knocking out of the Meq-cluster miRNAs significantly decreased the virus replication while deletion of the mid-cluster miRNAs had an enhancing effect on the virus replication. This was very different to that of the unchanged in vitro replication curves of the similar miR-KO GX0101 mutants produced by BAC mutagenesis, but completely in accordance with their in vivo replication and pathogenicity profiles in virus-challenged birds [27,28]. To further confirm that the observed changes in the virus growth kinetics, especially the improved proliferation of miR-M11-KO virus, were actually caused by the CRISPR/Cas9-mediated miRNA-deletion, we performed a compensatory overexpression of miR-M11 in CEFs in the present study. Our data have shown that the overexpression of miR-M11 successfully suppressed the enhanced growth of RB-1B∆M11 virus, strongly supporting our previous findings [28]. Whether miR-M11 directly regulates MDV genes to affect virus proliferation or through targeting host cellular genes to regulate cell proliferation and/or apoptosis deserves to be studied. In the previous studies using infectious BAC clones, it was suggested that the additional full-length BAC sequences within the genome of reconstituted viruses may have influenced their replication and pathogenicity [60,66]. Thus, our present data generated using CRISPR/Cas9 editing system, where the manipulation of the miRNA clusters resulted in generation of mutant viruses, are without the disadvantages of cloned full-length viral genomes.

In summary, we have presently demonstrated the use of the CRISPR/Cas9 gene editing system as a simple, efficient and relatively nondisruptive method to manipulate herpesvirus genomes in virus-infected cell cultures for studying functions of small non-coding RNAs. In our recent studies [67,68], we have developed a series of MDV-transformed cell lines stably expressing Cas9 (MSB-1-Cas9 and HP8-Cas9) and demonstrated a gene editing strategy involving the transfection of synthetic gRNAs with a two-part guide RNA system, for the efficient deletion of miR-M4 from the integrated viral genomes, which confirmed that the viral miR-155 ortholog critical for the induction of MD lymphomas is not essential for the proliferation of transformed cell lines [68]. We also obtained data suggesting that exosomes can transfer viral miRNAs from MSB-1 cells to primary CEF cells to exert regulatory functions (manuscript in preparation). Whether the viral miRNAs are also involved in such exosome-mediated transfer to play critical roles in such important processes requires further study. Undoubtedly, the establishment of a CRISPR/Cas9-based gene editing platform for manipulating viral genomes both in lytically replicating cell culture systems and in the virally-transformed lymphoblastoid cells will contribute significantly toward gaining more insights into the biology of this major oncogenic herpesvirus and the molecular mechanisms of lymphomas that it induces in its natural avian hosts.

## Supplementary Materials

The following are available online at https://www.mdpi.com/1999-4915/12/4/466/s1, Figure S1: Sequence alignment and identification of the gRNA-mediated miRNA mutagenesis in RB-1B viral genome, Figure S2: Immunofluorescence assays for detecting the expressions of miRNA adjacent viral proteins in RB-1B mutant-infected CEF cells, Table S1: sgRNA targeting sites and PAM sequences of targeted MDV-1 miRNA genes, Table S2: Oligo nucleotides used for making sgRNA plasmid, Table S3: Primers used for PCR identification of the miRNA-deleted mutants, Table S4: Primers and probes used for real-time qPCR analysis.

## References

[1] Kozomara A., Birgaoanu M., Griffiths-Jones S. (2019). miRBase: From microRNA sequences to function. Nucleic Acids Res..

[2] Bartel D.P. (2009). MicroRNAs: Target recognition and regulatory functions. Cell.

[3] Bartel D.P. (2018). Metazoan MicroRNAs. Cell.

[4] Boss I.W., Plaisance K.B., Renne R. (2009). Role of virus-encoded microRNAs in herpesvirus biology. Trends Microbiol..

[5] Grundhoff A., Sullivan C.S. (2011). Virus-encoded microRNAs. Virology.

[6] Kincaid R.P., Sullivan C.S. (2012). Virus-encoded microRNAs: An overview and a look to the future. PLoS Pathog..

[7] Morgan R.W., Burnside J. (2011). Roles of avian herpesvirus microRNAs in infection, latency, and oncogenesis. Biochim. Biophys. Acta.

[8] Burnside J., Bernberg E., Anderson A., Lu C., Meyers B.C., Green P.J., Jain N., Isaacs G., Morgan R.W. (2006). Marek’s disease virus encodes MicroRNAs that map to meq and the latency-associated transcript. J. Virol..

[9] Waidner L.A., Morgan R.W., Anderson A.S., Bernberg E.L., Kamboj S., Garcia M., Riblet S.M., Ouyang M., Isaacs G.K., Markis M. (2009). MicroRNAs of Gallid and Meleagrid herpesviruses show generally conserved genomic locations and are virus-specific. Virology.

[10] Yao Y., Zhao Y., Xu H., Smith L.P., Lawrie C.H., Sewer A., Zavolan M., Nair V. (2007). Marek’s disease virus type 2 (MDV-2)-encoded microRNAs show no sequence conservation with those encoded by MDV-1. J. Virol..

[11] Yao Y., Zhao Y., Xu H., Smith L.P., Lawrie C.H., Watson M., Nair V. (2008). MicroRNA profile of Marek’s disease virus-transformed T-cell line MSB-1: Predominance of virus-encoded microRNAs. J. Virol..

[12] Yao Y., Zhao Y., Smith L.P., Watson M., Nair V. (2009). Novel microRNAs (miRNAs) encoded by herpesvirus of Turkeys: Evidence of miRNA evolution by duplication. J. Virol..

[13] Davison A.J., Eberle R., Ehlers B., Hayward G.S., McGeoch D.J., Minson A.C., Pellett P.E., Roizman B., Studdert M.J., Thiry E. (2009). The order Herpesvirales. Arch. Virol..

[14] Payne L.N., Venugopal K. (2000). Neoplastic diseases: Marek’s disease, avian leukosis and reticuloendotheliosis. Rev. Sci. Tech..

[15] Osterrieder N., Kamil J.P., Schumacher D., Tischer B.K., Trapp S. (2006). Marek’s disease virus: From miasma to model. Nat. Rev. Microbiol..

[16] Jarosinski K.W., Tischer B.K., Trapp S., Osterrieder N. (2006). Marek’s disease virus: Lytic replication, oncogenesis and control. Expert Rev. Vaccines.

[17] Luo J., Teng M., Fan J., Wang F., Zhou L., Deng R., Zhang G. (2010). Marek’s disease virus-encoded microRNAs: Genomics, expression and function. Sci. China Life Sci..

[18] Zhao Y., Yao Y., Xu H., Lambeth L., Smith L.P., Kgosana L., Wang X., Nair V. (2009). A functional MicroRNA-155 ortholog encoded by the oncogenic Marek’s disease virus. J. Virol..

[19] Muylkens B., Coupeau D., Dambrine G., Trapp S., Rasschaert D. (2010). Marek’s disease virus microRNA designated Mdv1-pre-miR-M4 targets both cellular and viral genes. Arch. Virol..

[20] Zhao Y., Xu H., Yao Y., Smith L.P., Kgosana L., Green J., Petherbridge L., Baigent S.J., Nair V. (2011). Critical role of the virus-encoded microRNA-155 ortholog in the induction of Marek’s disease lymphomas. PLoS Pathog..

[21] Yu Z.H., Teng M., Sun A.J., Yu L.L., Hu B., Qu L.H., Ding K., Cheng X.C., Liu J.X., Cui Z.Z. (2014). Virus-encoded miR-155 ortholog is an important potential regulator but not essential for the development of lymphomas induced by very virulent Marek’s disease virus. Virology.

[22] Chi J.Q., Teng M., Yu Z.H., Xu H., Su J.W., Zhao P., Xing G.X., Liang H.D., Deng R.G., Qu L.H. (2015). Marek’s disease virus-encoded analog of microRNA-155 activates the oncogene c-Myc by targeting LTBP1 and suppressing the TGF-beta signaling pathway. Virology.

[23] Dang L., Teng M., Li H.Z., Ma S.M., Lu Q.X., Hao H.F., Zhao D., Zhou E.M., Zhang G.P., Luo J. (2017). Marek’s disease virus type 1 encoded analog of miR-155 promotes proliferation of chicken embryo fibroblast and DF-1 cells by targeting hnRNPAB. Vet. Microbiol..

[24] Zhuang G., Sun A., Teng M., Luo J. (2017). A Tiny RNA that Packs a Big Punch: The Critical Role of a Viral miR-155 Ortholog in Lymphomagenesis in Marek’s Disease. Front. Microbiol..

[25] Xu S., Xue C., Li J., Bi Y., Cao Y. (2011). Marek’s disease virus type 1 microRNA miR-M3 suppresses cisplatin-induced apoptosis by targeting Smad2 of the transforming growth factor beta signal pathway. J. Virol..

[26] Strassheim S., Stik G., Rasschaert D., Laurent S. (2012). mdv1-miR-M7-5p, located in the newly identified first intron of the latency-associated transcript of Marek’s disease virus, targets the immediate-early genes ICP4 and ICP27. J. Gen. Virol..

[27] Teng M., Yu Z.H., Sun A.J., Min Y.J., Chi J.Q., Zhao P., Su J.W., Cui Z.Z., Zhang G.P., Luo J. (2015). The significance of the individual Meq-clustered miRNAs of Marek’s disease virus in oncogenesis. J. Gen. Virol..

[28] Teng M., Yu Z.H., Zhao P., Zhuang G.Q., Wu Z.X., Dang L., Li H.Z., Ma S.M., Cui Z.Z., Zhang G.P. (2017). Putative roles as oncogene or tumour suppressor of the Mid-clustered microRNAs in Gallid alphaherpesvirus 2 (GaHV2) induced Marek’s disease lymphomagenesis. J. Gen. Virol..

[29] Brouns S.J., Jore M.M., Lundgren M., Westra E.R., Slijkhuis R.J., Snijders A.P., Dickman M.J., Makarova K.S., Koonin E.V., van der Oost J. (2008). Small CRISPR RNAs guide antiviral defense in prokaryotes. Science.

[30] Jansen R., Embden J.D., Gaastra W., Schouls L.M. (2002). Identification of genes that are associated with DNA repeats in prokaryotes. Mol. Microbiol..

[31] Jinek M., Chylinski K., Fonfara I., Hauer M., Doudna J.A., Charpentier E. (2012). A programmable dual-RNA-guided DNA endonuclease in adaptive bacterial immunity. Science.

[32] Van der Oost J., Jore M.M., Westra E.R., Lundgren M., Brouns S.J. (2009). CRISPR-based adaptive and heritable immunity in prokaryotes. Trends Biochem. Sci..

[33] Cong L., Ran F.A., Cox D., Lin S., Barretto R., Habib N., Hsu P.D., Wu X., Jiang W., Marraffini L.A. (2013). Multiplex genome engineering using CRISPR/Cas systems. Science.

[34] Hsu P.D., Lander E.S., Zhang F. (2014). Development and applications of CRISPR-Cas9 for genome engineering. Cell.

[35] Mali P., Esvelt K.M., Church G.M. (2013). Cas9 as a versatile tool for engineering biology. Nat. Methods.

[36] Yuen K.S., Chan C.P., Wong N.M., Ho C.H., Ho T.H., Lei T., Deng W., Tsao S.W., Chen H., Kok K.H. (2015). CRISPR/Cas9-mediated genome editing of Epstein-Barr virus in human cells. J. Gen. Virol..

[37] Suenaga T., Kohyama M., Hirayasu K., Arase H. (2014). Engineering large viral DNA genomes using the CRISPR-Cas9 system. Microbiol. Immunol..

[38] Bi Y., Sun L., Gao D., Ding C., Li Z., Li Y., Cun W., Li Q. (2014). High-efficiency targeted editing of large viral genomes by RNA-guided nucleases. PLoS Pathog..

[39] Bierle C.J., Anderholm K.M., Wang J.B., McVoy M.A., Schleiss M.R. (2016). Targeted Mutagenesis of Guinea Pig Cytomegalovirus Using CRISPR/Cas9-Mediated Gene Editing. J. Virol..

[40] Peng Z., Ouyang T., Pang D., Ma T., Chen X., Guo N., Chen F., Yuan L., Ouyang H., Ren L. (2016). Pseudorabies virus can escape from CRISPR-Cas9-mediated inhibition. Virus Res..

[41] Tang Y.D., Liu J.T., Wang T.Y., An T.Q., Sun M.X., Wang S.J., Fang Q.Q., Hou L.L., Tian Z.J., Cai X.H. (2016). Live attenuated pseudorabies virus developed using the CRISPR/Cas9 system. Virus Res..

[42] Xu A., Qin C., Lang Y., Wang M., Lin M., Li C., Zhang R., Tang J. (2015). A simple and rapid approach to manipulate pseudorabies virus genome by CRISPR/Cas9 system. Biotechnol. Lett..

[43] Liang X., Sun L., Yu T., Pan Y., Wang D., Hu X., Fu Z., He Q., Cao G. (2016). A CRISPR/Cas9 and Cre/Lox system-based express vaccine development strategy against re-emerging Pseudorabies virus. Sci. Rep..

[44] Yuan M., Zhang W., Wang J., Al Yaghchi C., Ahmed J., Chard L., Lemoine N.R., Wang Y. (2015). Efficiently editing the vaccinia virus genome by using the CRISPR-Cas9 system. J. Virol..

[45] Chang P., Yao Y., Tang N., Sadeyen J.R., Sealy J., Clements A., Bhat S., Munir M., Bryant J.E., Iqbal M. (2018). The Application of NHEJ-CRISPR/Cas9 and Cre-Lox System in the Generation of Bivalent Duck Enteritis Virus Vaccine against Avian Influenza Virus. Viruses.

[46] Zou H., Su R., Ruan J., Shao H., Qian K., Ye J., Yao Y., Nair V., Qin A. (2017). Double-stranded RNA induces chicken T-cell lymphoma apoptosis by TRIF and NF-kappaB. Sci. Rep..

[47] Yao Y., Bassett A., Nair V. (2016). Targeted editing of avian herpesvirus vaccine vector using CRISPR/Cas9 nucleases. J. Vaccine Technol..

[48] Tang N., Zhang Y., Pedrera M., Chang P., Baigent S., Moffat K., Shen Z., Nair V., Yao Y. (2018). A simple and rapid approach to develop recombinant avian herpesvirus vectored vaccines using CRISPR/Cas9 system. Vaccine.

[49] Zhang Y., Tang N., Sadigh Y., Baigent S., Shen Z., Nair V., Yao Y. (2018). Application of CRISPR/Cas9 Gene Editing System on MDV-1 Genome for the Study of Gene Function. Viruses.

[50] Tang N., Zhang Y., Pedrera M., Chang P., Baigent S., Moffat K., Shen Z., Nair V., Yao Y. (2019). Generating Recombinant Avian Herpesvirus Vectors with CRISPR/Cas9 Gene Editing. J. Vis. Exp..

[51] Tang N., Zhang Y., Sadigh Y., Moffat K., Shen Z., Nair V., Yao Y. (2020). Generation of A Triple Insert Live Avian Herpesvirus Vectored Vaccine Using CRISPR/Cas9-Based Gene Editing. Vaccines.

[52] Ran F.A., Hsu P.D., Wright J., Agarwala V., Scott D.A., Zhang F. (2013). Genome engineering using the CRISPR-Cas9 system. Nat. Protoc..

[53] Labun K., Montague T.G., Krause M., Torres Cleuren Y.N., Tjeldnes H., Valen E. (2019). CHOPCHOP v3: Expanding the CRISPR web toolbox beyond genome editing. Nucleic Acids Res..

[54] Baigent S.J., Smith L.P., Currie R.J.W., Nair V.K. (2005). Replication kinetics of Marek’s disease vaccine virus in feathers and lymphoid tissues using PCR and virus isolation. J. Gen. Virol..

[55] Baigent S.J., Smith L.P., Petherbridge L.J., Nair V.K. (2011). Differential quantification of cloned CVI988 vaccine strain and virulent RB-1B strain of Marek’s disease viruses in chicken tissues, using real-time PCR. Res. Vet. Sci..

[56] Bell E.J., Brickell P.M. (1997). Replication-competent retroviral vectors for expressing genes in avian cells in vitro and in vivo. Mol. Biotechnol..

[57] Muyrers J.P., Zhang Y., Testa G., Stewart A.F. (1999). Rapid modification of bacterial artificial chromosomes by ET-recombination. Nucleic Acids Res..

[58] Narayanan K., Williamson R., Zhang Y., Stewart A.F., Ioannou P.A. (1999). Efficient and precise engineering of a 200 kb beta-globin human/bacterial artificial chromosome in *E. coli* DH10B using an inducible homologous recombination system. Gene. Ther..

[59] Petherbridge L., Howes K., Baigent S.J., Sacco M.A., Evans S., Osterrieder N., Nair V. (2003). Replication-competent bacterial artificial chromosomes of Marek’s disease virus: Novel tools for generation of molecularly defined herpesvirus vaccines. J. Virol..

[60] Petherbridge L., Brown A.C., Baigent S.J., Howes K., Sacco M.A., Osterrieder N., Nair V.K. (2004). Oncogenicity of virulent Marek’s disease virus cloned as bacterial artificial chromosomes. J. Virol..

[61] Schumacher D., Tischer B.K., Fuchs W., Osterrieder N. (2000). Reconstitution of Marek’s disease virus serotype 1 (MDV-1) from DNA cloned as a bacterial artificial chromosome and characterization of a glycoprotein B-negative MDV-1 mutant. J. Virol..

[62] Sun A.J., Xu X.Y., Petherbridge L., Zhao Y.G., Nair V., Cui Z.Z. (2010). Functional evaluation of the role of reticuloendotheliosis virus long terminal repeat (LTR) integrated into the genome of a field strain of Marek’s disease virus. Virology.

[63] Coupeau D., Dambrine G., Rasschaert D. (2012). Kinetic expression analysis of the cluster mdv1-mir-M9-M4, genes meq and vIL-8 differs between the lytic and latent phases of Marek’s disease virus infection. J. Gen. Virol..

[64] Luo J., Sun A.J., Teng M., Zhou H., Cui Z.Z., Qu L.H., Zhang G.P. (2011). Expression profiles of microRNAs encoded by the oncogenic Marek’s disease virus reveal two distinct expression patterns in vivo during different phases of disease. J. Gen. Virol..

[65] Zhao P., Li X.J., Teng M., Dang L., Yu Z.H., Chi J.Q., Su J.W., Zhang G.P., Luo J. (2015). In vivo expression patterns of microRNAs of Gallid herpesvirus 2 (GaHV-2) during the virus life cycle and development of Marek’s disease lymphomas. Virus Genes.

[66] Zhao Y., Petherbridge L., Smith L.P., Baigent S., Nair V. (2008). Self-excision of the BAC sequences from the recombinant Marek’s disease virus genome increases replication and pathogenicity. Virol. J..

[67] Zhang Y., Luo J., Tang N., Teng M., Reddy V., Moffat K., Shen Z., Nair V., Yao Y. (2019). Targeted Editing of the pp38 Gene in Marek’s Disease Virus-Transformed Cell Lines Using CRISPR/Cas9 System. Viruses.

[68] Zhang Y., Tang N., Luo J., Teng M., Moffat K., Shen Z., Watson M., Nair V., Yao Y. (2019). Marek’s disease virus-encoded microRNA 155 ortholog critical for the induction of lymphomas is not essential for the proliferation of transformed cell Lines. J. Virol..

