# Supplementary material for: Efficient Mutagenesis of Marek’s Disease Virus-Encoded microRNAs Using a CRISPR/Cas9-Based Gene Editing System
Source: Viruses. 2020 Apr 20;12(4):466. doi: 10.3390/v12040466 (PMC7232411; doi:10.3390/v12040466)
Supplement: Supplementary file 1 [file viruses-12-00466-s001.pdf]

Supplemental Tables:

Table S1. sgRNA targeting sites and PAM sequences of targeted MDV-1 miRNA genes.

Target miRNA	SgRNA name	Target sequence (5'-3') *	Sense/antisense	Score	Genomic location
**miR-M9**	M9gR1	CTCCGGGAGTGTAACATATC(GGG)	+	91	upstream to miR-M9-5p
	M9gR2	TCATATACATTTCCGTACGC(GGG)	+	98	upstream to miR-M9-5p
	M9gR3	TCGTACGTTGTAAACTCCGA(GGG)	+	98	inside of miR-M9-3p
**miR-M4**	M4gR916	CTGTATCGGAACCCTTCGTT(CGG)	+	97	inside of miR-M4-5p
	M4gR917	CGTGTTCCACGTGACGGCTC(TGG)	+	96	upstream to miR-M4-5p
**miR-M11**	M11gR1	CGAGTCTAAGCTACACGGTA(AGG)	-	98	inside of miR-M11-5p
	M11gR2	CTTACCGTGTAGCTTAGACT(CGG)	+	92	inside of miR-M11-5p
	M11gR3	TCTTCCGAGTCTAAGCTACA(CGG)	-	92	inside of miR-M11-5p
	M11gR4	CGTTCTATACAGAACGGTTG(GGG)	+	95	downstream to miR-M11-3p
	M11gR5	ATGGAGTATTATTCGGGATA(TGG)	+	95	downstream to miR-M11-3p
	M11gR6	GATATGGACATCGCACATTA(AGG)	+	92	downstream to miR-M11-3p
	M11gR7	GTGCTGATTAGGTTATCGTA(AGG)	+	94	downstream to miR-M11-3p
	M11gR8	TCATCCGCGTCCCAGCAATC(AGG)	+	92	downstream to miR-M11-3p
**miR-M1**	M1gR1	ATGAAAGAGCGAACGGAACG(AGG)	+	93	downstream to miR-M1-3p
	M1gR2	GCTGCGCATGAAAGAGCGAA(CGG)	+	90	downstream to miR-M1-3p

* The protospacer adjacent motif (PAM) is shown in brackets.

Table S2. Oligo nucleotides used for making sgRNA plasmids.

Target miRNA	SgRNA name	Oligo nucleotide name	Sense/antisense	Length (nt)	Sequence (5'-3') *
**miR-M9**	M9gR1	M9gR1-5p	+	25	CACCGCTCCGGGAGTGTAACATATC
		M9gR1-3p	-	25	AAACGATATGTTACACTCCCGGAGC
	M9gR2	M9gR2-5p	+	25	CACCGTCATATACATTTCCGTACGC
		M9gR2-3p	-	25	AAACGCGTACGGAAATGTATATGAC
	M9gR3	M9gR3-5p	+	25	CACCGTCGTACGTTGTAAACTCCGA
		M9gR3-3p	-	25	AAACTCGGAGTTTACAACGTACGAC
**miR-M4**	M4gR916	M4gR916-5p	+	24	CACCTGTATCGGAACCCTTCGTT
		M4gR916-3p	-	24	AAACAACGAAGGGTTCCGATACA
	M4gR917	M4gR917-5p	+	24	CACCCGTGTTCCACGTGACGGCTC
		M4gR917-3p	-	24	AAACGAGCCGTCACGTGGAACACG
**miR-M11**	M11gR1	M11gR1-5p	+	25	CACCGCGAGTCTAAGCTACACGGTA
		M11gR1-3p	-	25	AAACTACCGTGTAGCTTAGACTCGC
	M11gR2	M11gR2-5p	+	25	CACCGCTTACCGTGTAGCTTAGACT
		M11gR2-3p	-	25	AAACAGTCTAAGCTACACGGTAAGC
	M11gR3	M11gR3-5p	+	25	CACCGTCTTCCGAGTCTAAGCTACA
		M11gR3-3p	-	25	AAACTGTAGCTTAGACTCGGAAGAC
	M11gR4	M11gR4-5p	+	25	CACCGCGTTCTATACAGAACGGTTG
		M11gR4-3p	-	25	AAACCAACCGTTCTGTATAGAACGC
	M11gR5	M11gR5-5p	+	25	CACCGATGGAGTATTATTCGGGATA
		M11gR5-3p	-	25	AAACTATCCCGAATAATACTCCATC
	M11gR6	M11gR6-5p	+	24	CACCGATATGGACATCGCACATTA
		M11gR6-3p	-	24	AAACTAATGTGCGATGTCCATATC
	M11gR7	M11gR7-5p	+	24	CACCGTGCTGATTAGGTTATCGTA
		M11gR7-3p	-	24	AAACTACGATAACCTAATCAGCAC
	M11gR8	M11gR8-5p	+	25	CACCGTCATCCGCGTCCCAGCAATC
		M11gR8-3p	-	25	AAACGATTGCTGGGACGCGGATGAC
**miR-M1**	M1gR1	M1gR1-5p	+	25	CACCGATGAAAGAGCGAACGGAACG
		M1gR1-3p	-	25	AAACCGTTCCGTTCGCTCTTTCATC
	M1gR2	M1gR2-5p	+	24	CACCGCTGCGCATGAAAGAGCGAA
		M1gR2-3p	-	24	AAACTTCGCTCTTTCATGCGCAGC

* The flanked restriction enzyme sites of *Bbs* I are shown in red and the additionally added G or C ahead of gRNA are shown in blue.

Table S3. Primers used for PCR identification of the miRNA-deleted mutants.

Primer pair	Primer name	Type	Length (nt)	Sequence (5'-3')	wt/mut amplicons (bp)	Usage
1	Meq5F-133168	5'	22	5'-CCAAAGTGCGGGTAAGGTAATC-3'	1427/407 or	Meq-miRs or miR-
	Meq3R-134595	3'	22	5'-GTATCACTCCCGAACCATTAGA-3'	1427/347	M9-M2
**2**	Meq5F-133168	5'	22	5'-CCAAAGTGCGGGTAAGGTAATC-3'	644/507	miR-M9
	M5-3R-133811	3'	20	5'-CCATATCTCAAACAACGCGA-3'		
**3**	miRM4_F	5'	19	5'-TGAGGGGAGCGATCGACTC-3'	205/150	miR-M4
	miRM4_R	3'	26	5'-GATTCAATATTACATCACTTCAACGG-3'		
**4**	Mid5F-135890	5'	22	5'-TCCGCATTGTGACTCTCAGCAG-3'	1135/835	miR-M11
	Mid3R-137024	3'	22	5'-ACATCGTAGAGAAAGCATAAGG-3'		

Table S4. Primers and probes used for real-time qPCR analysis.

Primer pair	Assay name	Type	Assay ID	Product Lot No. *
**1**	mdv1-miR-M4	RT primer	RT: 007758 mat	P131213-000 C10
		TM probe	TM: 007758 mat	P131213-000 C10
**2**	mdv1-miR-M9	RT primer	RT: 007758 mat	P131213-000 C05
		TM probe	TM: 007758 mat	P131213-000 C05
**3**	mdv1-miR-M11-5p	RT primer	RT: 007758 mat	P131213-000 C11
		TM probe	TM: 007758 mat	P131213-000 C11
**4**	mdv1-miR-M12	RT primer	RT: 007758 mat	P131213-000 C07
		TM probe	TM: 007758 mat	P131213-000 C07
**5**	mdv1-miR-M31	RT primer	RT: 007758 mat	P131213-000 C12
		TM probe	TM: 007758 mat	P131213-000 C12

* Provided by Applied Biosystems, Thermo Fisher Scientific.

Supplemental Figures & Legends:

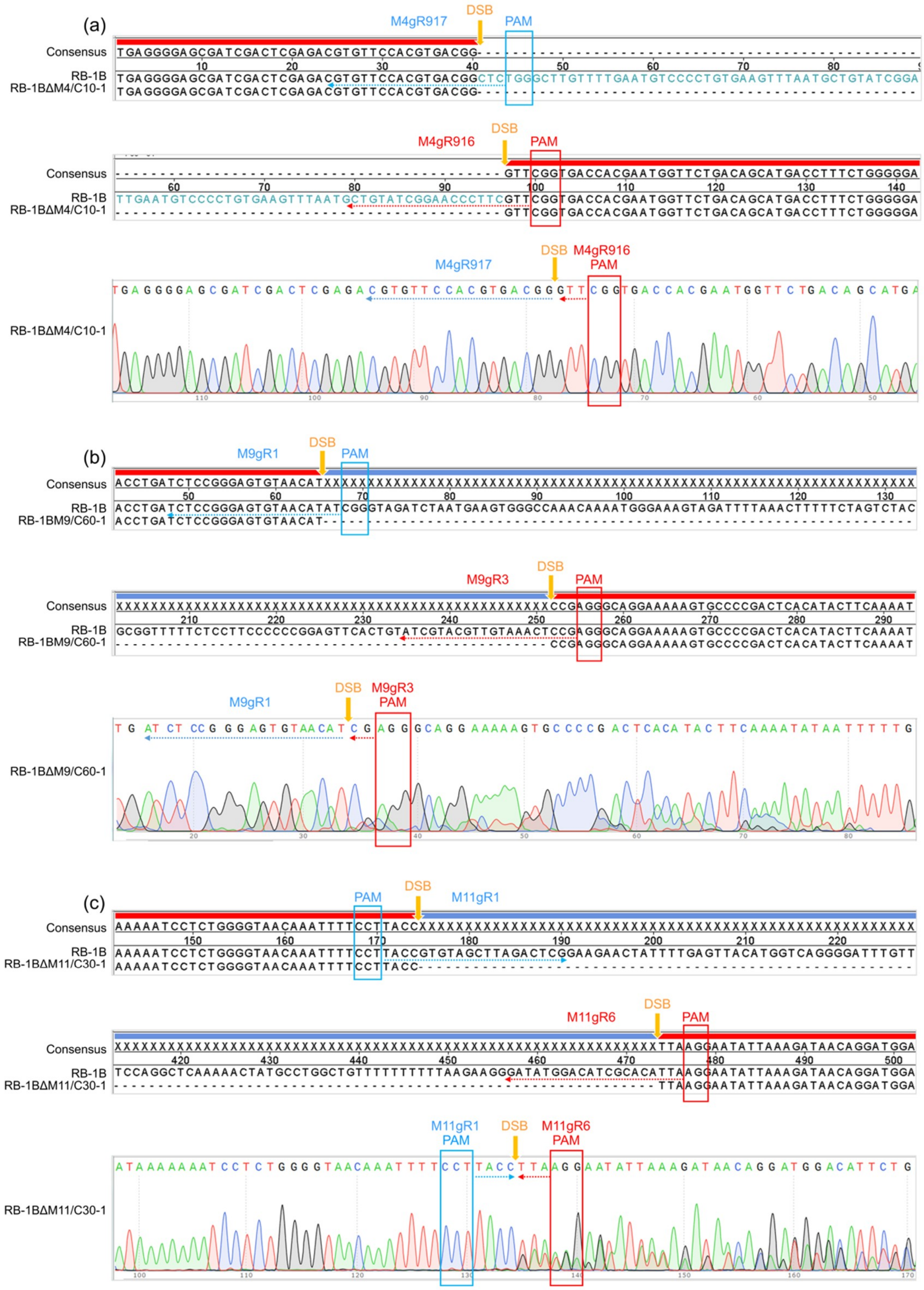

Figure S1. Sequence alignment and identification of the gRNA-mediated miRNA mutagenesis in RB-1B viral genome. (a, b & c) RB-1B mutants with the deletions of miR-M4, miR-M9 or miR-M11. The double strand breaks (DSBs) in targeted miRNA genes are shown by yellow arrows. The entire or broken gRNA sequences and protospacer adjacent motifs (PAMs) are shown by same colored arrows or square frames, respectively.

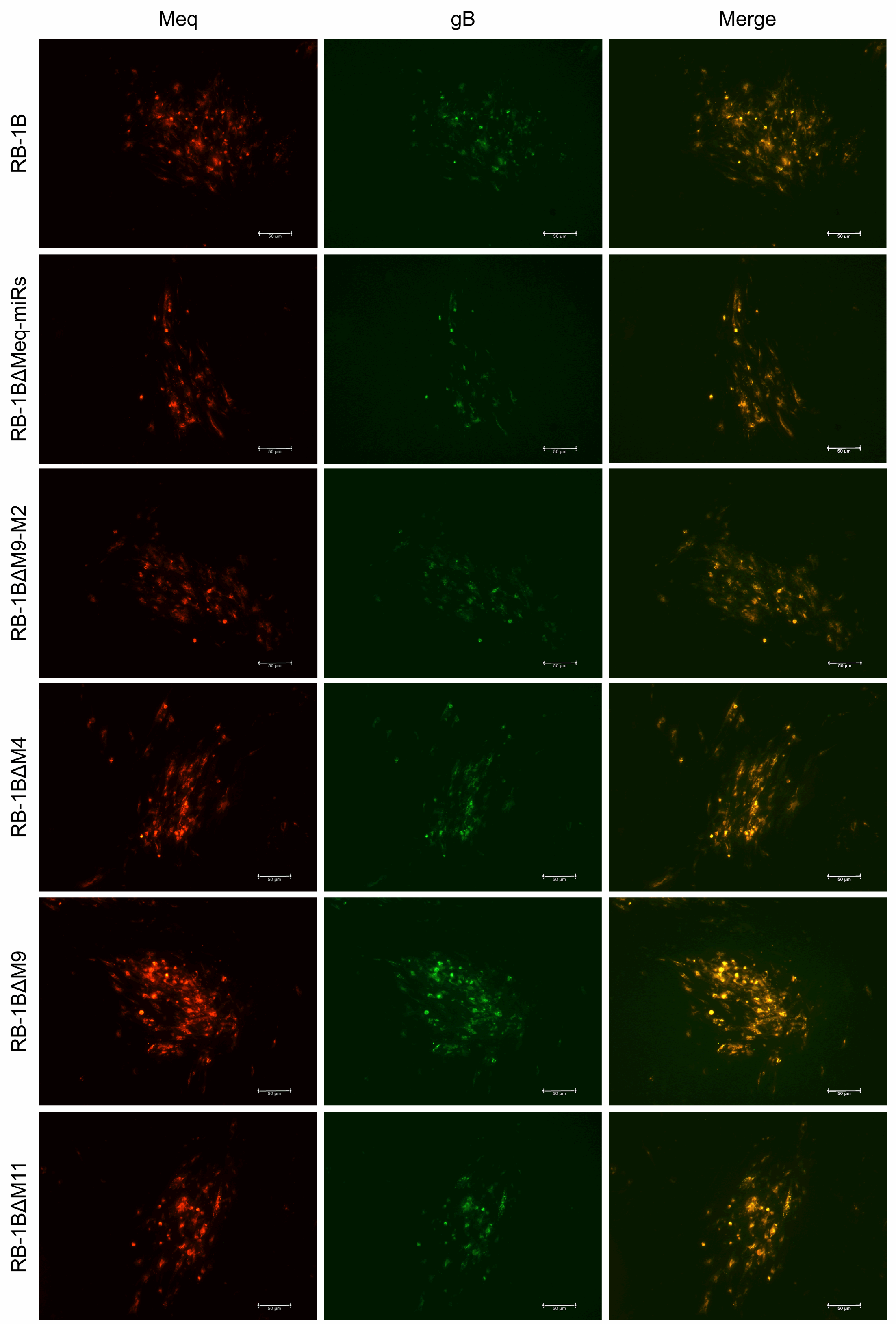

Figure 2. Immunofluorescence assays for detecting the expressions of miRNA adjacent viral proteins in RB-1B mutant-infected CEF cells. Meq, Marek's EcoQ-encoded protein; gB, glycoprotein B. Scale bar = 50 μm.
